# Pretreatment Quality of Life and Substance Use Among Patients Diagnosed With Head and Neck Cancer

**DOI:** 10.1002/cam4.70399

**Published:** 2024-11-08

**Authors:** Eric Adjei Boakye, Sami I. Nassar, Suma J. Alzouhayli, Amy M. Williams, Steven S. Chang, Tamer A. Ghanem, Marissa Gilbert, Suhael Momin, Farzan Siddiqui, Vivian F. Wu, Samantha H. Tam

**Affiliations:** ^1^ Department of Epidemiology and Biostatistics Michigan State University College of Human Medicine East Lansing Michigan USA; ^2^ Department of Otolaryngology—Head & Neck Surgery Henry Ford Health System Detroit Michigan USA; ^3^ Department of Public Health Sciences Henry Ford Health System Detroit Michigan USA; ^4^ Department of Otolaryngology—Head and Neck Surgery Medical University of South Carolina Charleston South Carolina USA; ^5^ Department of Surgery Wayne State University School of Medicine Detroit Michigan USA; ^6^ Office of Physician Wellness Henry Ford Health System Detroit Michigan USA; ^7^ Premier Head and Neck Surgery PC Flint Michigan USA; ^8^ Department of Radiation Oncology Henry Ford Health System Detroit Michigan USA

**Keywords:** cannabis use, FACT‐HN, head and neck cancer, illicit drug use, quality of life, substance abuse

## Abstract

**Background:**

There is a paucity of research on the effects of commonly used substances, such as cannabis and other drugs, on quality of life as a contributor to head and neck cancer (HNC) prognosis. We examined associations between non‐alcohol or tobacco substance use (cannabis and other illicit drug) and self‐reported quality of life in patients with HNC prior to starting treatment.

**Methods:**

This was a cross‐sectional study of patients who presented for routine psych‐oncologevaluation prior to treatment between 11/2015 and 9/2022. Primary exposures were cannabis use (never, past, or current users) and current illicit drug use (yes/no). The primary outcome measure was the Functional Assessment of Cancer Therapy—Head and Neck (FACT‐HN) subscales (physical, social/family, functional and emotional). Linear regression models examined associations between pretreatment substance use and FACT‐HN subscales adjusting for demographic, socioeconomic, and clinical factors.

**Results:**

Of 570 patients, 13.9% endorsed current cannabis and 13.9% current illicit drug use. The mean (SD) scores for FACT‐HN subscales were physical well‐being = 22.8 (5.0), social well‐being = 22.7 (5.5), emotional well‐being = 17.5 (4.5), and functional well‐being = 18.7 (6.9). In the adjusted models, cannabis use was not independently associated with any FACT‐HN subscales. However, patients who currently used illicit drugs reported worse emotional well‐being (*β* = −1.32; 95% CI −2.45 to −0.20). No independent association was found between current illicit drug use and other subscales (physical, social, and functional).

**Conclusions:**

Illicit drug use, but not cannabis use, is negatively associated with pretreatment emotional well‐being in patients with HNC. Further research exploring the relationships between longitudinal cannabis and illicit drug use and methods of consumption on QoL and cancer outcomes in patients with head and neck cancer is warranted.

## 1 Introduction

Head and neck cancer (HNC) is associated with a decrease in patients' quality of life (QoL), both before and after treatment [1, 2]. Factors that contribute to lower QoL among patients with HNC include disturbances related to speaking, eating, breathing, facial disfigurement, and pain, all of which negatively affect patients' social, physical, emotional, and cognitive functioning [2, 3, 4]. Pain is reported in up to 70% of patients with HNC, which is the highest prevalence when compared to patients diagnosed with other cancers [4].

Tobacco and alcohol consumption are two main risk factors for HNC, with use of each being associated with up to 10 and 13 times increased risk of developing HNC, respectively [5, 6]. There is a high prevalence of continued tobacco and alcohol use in patients with HNC following diagnosis, which is associated with poorer QoL [1, 3, 7, 8, 9]. However, substance use among patients with HNC following diagnosis but prior to commencing treatment is less frequently characterized and studied. Independent of HNC, substance use, and substance use disorders are associated with low QoL, while treatment of substance use disorder leads to improved QoL [10].

The influence of tobacco and/or alcohol use on QoL and HNC outcomes can inform management decisions in HNC care. Given the prominent role of tobacco and alcohol in head and neck carcinogenesis and QoL, there is a paucity of research on the effects of other commonly used substances, such as cannabis and other drugs, on QoL in HNC. This study aims to contribute to this body of literature by examining the association between cannabis use and other illicit drug use (cocaine, heroin, or prescription medication misuse), and self‐reported QoL in patients with HNC in the pre‐treatment initiation period.

## 2 Materials and Methods

### 2.1 Study Design, Setting, Sample and Data Collection

This study was approved by the Institutional Review Board of Henry Ford Hospital in Detroit, Michigan. In this cross‐sectional study, adult patients diagnosed with HNC between November 2015 and September 2022 who presented for treatment and management at a single urban tertiary care institution were included. Patients routinely underwent an evaluation with psych‐oncology prior to treatment as a part of usual care for all patients with HNC. Based on the total volume of patients with HNC seen at our institution, approximately 96% are seen by psych‐oncology prior to commencing treatment. Only patients who had completed this pretreatment evaluation with psych‐oncology were included in the study. The Functional Assessment of Cancer Therapy—Head and Neck (FACT‐HN) Version 4 [11] was completed by the patient via computer or paper format and the patient engaged in a standardized clinical interview with the psych‐oncologist. During the standardized clinical interview, which is part of routine clinical care at our institution, all patients are asked every question in the same order by the psych‐oncology team. The psych‐oncology team consists of one supervising psych‐oncologist and their post‐doctoral fellow, all of whom are trained to deliver the same standardized interview to each patient. The standardized interview queried social, psychological, and substance use factors (e.g., How often do you drink alcohol? On a day that you are drinking, how much do you typically drink? What is the size of these drinks?). Data from these interviews were retrospectively chart reviewed, extracted from the electronic medical records of participating patients, and recorded into a digital database of patients receiving treatment at the institution.

### 2.2 Measures

The primary outcome measurement for the study was the FACT‐HN questionnaire [11]. The FACT‐HN is a multidimensional, self‐reported QoL instrument specifically designed for use in patients with HNC. The FACT‐HN is comprised of 27 items encompassing four domains of well‐being: physical, social/family, functional (each with seven questions, range 0–28), emotional (six questions, range 0–24), and a fifth subscale of 12 additional items (range 0–40) assessing head and neck‐related symptoms; of note, item code HN 8 and 9 are not scored [11]. Each subscale is comprised of statements that patients rank on a five‐point Likert scale to indicate the degree of the statement's applicability over the past 7 days. Each item was scored from 0 to 4 and then item scores were combined to produce subscale scores for each domain. Higher scores represent better QoL.

The main exposure variables were cannabis use and other current illicit drug use (including cocaine, heroin, or prescription drug misuse). This information was obtained through the standardized clinical interview with the psych‐oncology team. Cannabis use was categorized as never, former, or current use and current illicit drug use was categorized as (yes/no).

Covariates included were age (years), gender (male/female), marital status (married/partnered, separated/divorced/widowed, single), education level (college graduate or higher, some college/associate degree, high school diploma/GED, no high school diploma), insurance type (Private, Medicare, Medicaid/other), tobacco use status (never, former, current), alcohol use (none/social use, former heavy use, current heavy use), number of comorbidities based on the Charlson Comorbidity Index (zero, one, ≥ two), and anatomic site (Oropharynx, Larynx, Oral cavity, other sites (including unknown primary)/missing).

### 2.3 Statistical Analysis

Descriptive statistics (e.g., frequencies for categorical variables and mean [SD] for continuous variables) were used to characterize the study sample. Patient characteristics (sociodemographic, behavioral, and clinical) were compared with cannabis use using chi‐square tests and one‐way ANOVA where appropriate. Five multivariable linear regression models were used to examine the association between cannabis use and each of the five FACT‐HN subscales controlling for the model and adjusting for age, gender, race, marital status, education level, insurance type, tobacco use, alcohol use, comorbidities, and anatomic site. Similarly, five multivariable linear regression models were used to examine the association between current illicit drug use and the five FACT‐HN subscales adjusting for the same variables previously stated. A two‐sided *p* < 0.05 was considered statistically significant for all variables. Statistical analyses were performed using SAS statistical software version 9.4 (SAS Institute Inc. Cary NC).

## 3 Results

A total of 570 patients were included in the study. The mean age of patients at time of initial diagnosis was 62.2 years (SD = 11.4). Patient characteristics are presented in Table 1. The majority of the study sample were male (68.6%) and White (71.9%). About one‐third (35.2%) of the patients currently used tobacco, 23.9% reported current/frequent alcohol use, and 38.4% had two or more comorbidities. Approximately 14% of the patients indicated they currently used cannabis and 24% used cannabis in the past. Similarly, 13.9% reported currently using illicit drugs. The mean ± SD scores for FACT‐HN subscales were as follows: physical well‐being = 22.8 ± 5.0, social/familial well‐being = 22.7 ± 5.5, emotional well‐being = 17.5 ± 4.5, functional well‐being = 18.7 ± 6.9, and head and neck cancer‐related symptoms = 28.4 ± 8.2.

**TABLE 1:** Patient characteristics of the included cohort (*N* = 570).

	*N* (%)
Age (mean ± SD)	62.2 ± 11.5
< 65 years	341 (59.8)
≥ 65 years	229 (40.2)
Gender	
Female	179 (31.4)
Male	391 (68.6)
Race	
White	410 (71.9)
Other races	160 (28.1)
Marital status	
Married/partnered	272 (47.7)
Separated/divorced/widowed	197 (34.6)
Single	101 (17.7)
Education level	
College graduate or higher	155 (27.2)
Some college/associate degree	159 (27.9)
High school diploma	155 (27.2)
< High school diploma	101 (17.7)
Insurance type	
Private	173 (30.3)
Medicare	148 (26.0)
Medicaid/other	249 (43.7)
Smoking status	
Never	156 (27.4)
Former	213 (37.4)
Current	201 (35.2)
Alcohol use	
No/social drinker	222 (38.9)
Former/occasional drinker	212 (37.2)
Current/frequent drinker	136 (23.9)
Comorbidities	
Zero	226 (39.7)
One	125 (21.9)
≥ Two	219 (38.4)
Anatomic site	
Oropharynx	93 (16.3)
Larynx	63 (11.1)
Oral cavity	89 (15.6)
Other/missing	325 (57.0)
Cannabis use	
Never	252 (44.2)
Past	136 (23.8)
Current	79 (13.9)
Missing	103 (18.1)
Current illicit drug use	
Yes	79 (13.9)
No	386 (67.7)
Missing	105 (18.4)
	**Mean SD**
Physical well‐being	22.8 ± 5.0
Social well‐being	22.7 ± 5.5
Emotional well‐being	17.5 ± 4.5
Functional well‐being	18.7 ± 6.9
Head and neck cancer	28.4 ± 8.2
Total FACT‐HN score	111.0 ± 23.6

When patient characteristics were stratified by cannabis use in the univariate analysis, age, gender, race, marital status, education level, insurance type, tobacco use, alcohol use, and anatomic site were significantly associated with cannabis use (Table 2). Patients who reported current cannabis use were more likely to be younger, male, White, single/unpartnered, have a high school diploma, and currently use tobacco (Table 2). For FACT‐HN subscales, only emotional well‐being was significantly associated with current cannabis use on univariate analysis (Table 2).

**TABLE 2:** Patient characteristics stratified by cannabis use.

	Mean ± SD				*p*
	Never	Past	Current	Missing	
Physical well‐being	22.9 ± 5.0	22.6 ± 5.0	22.1 ± 5.9	23.2 ± 4.4	0.4667
Social well‐being	23.0 ± 5.3	22.4 ± 5.6	22.1 ± 6.3	23.2 ± 5.1	0.4211
Emotional well‐being	18.1 ± 4.4	17.3 ± 4.5	16.8 ± 5.0	16.8 ± 4.4	0.0324
Functional well‐being	18.7 ± 7.0	18.5 ± 6.7	18.1 ± 7.6	19.4 ± 6.6	0.6523
Head and neck cancer	28.3 ± 8.5	28.3 ± 7.1	28.5 ± 9.3	28.6 ± 8.2	0.9870
	*N* (%)				
Age					
< 65 years	112 (32.8)	85 (24.9)	60 (17.6)	84 (24.6)	< 0.0001
≥ 65 years	140 (61.1)	51 (22.3)	19 (8.3)	19 (8.3)	
Gender					
Female	89 (49.7)	32 (17.9)	12 (6.7)	46 (25.7)	< 0.0001
Male	163 (41.7)	104 (26.6)	67 (17.1)	57 (14.6)	
Race					
White	194 (47.3)	102 (24.9)	61 (14.9)	53 (12.9)	< 0.0001
Other races	58 (36.3)	34 (21.3)	18 (11.2)	50 (31.2)	
Marital status					
Married/partnered	142 (52.2)	71 (26.1)	30 (11.0)	29 (10.7)	< 0.0001
Separated/divorced/widowed	65 (33.0)	42 (21.3)	28 (14.2)	62 (31.5)	
Single	45 (44.6)	23 (22.8)	21 (20.8)	12 (11.9)	
Education level					
College graduate or higher	73 (47.1)	27 (17.4)	23 (14.8)	32 (20.7)	< 0.0001
Some college/associate degree	78 (49.1)	48 (30.2)	23 (14.5)	10 (6.3)	
High school diploma	75 (48.4)	43 (27.7)	25 (16.1)	12 (7.7)	
< High school diploma	26 (25.7)	18 (17.8)	8 (7.9)	49 (48.5)	
Insurance type					
Private	83 (48.0)	46 (26.6)	27 (15.6)	17 (9.8)	< 0.0001
Medicare	66 (44.6)	47 (31.8)	13 (8.8)	22 (14.9)	
Medicaid/other	103 (41.4)	43 (17.3)	39 (15.7)	64 (25.7)	
Smoking status					
Never	98 (62.8)	24 (15.4)	17 (10.9)	17 (10.9)	< 0.0001
Former	103 (48.4)	58 (27.2)	26 (12.2)	26 (12.2)	
Current	51 (25.4)	54 (26.9)	36 (17.9)	60 (29.8)	
Alcohol use					
No/social drinker	134 (60.4)	43 (19.4)	19 (8.6)	26 (11.7)	< 0.0001
Former/occasional drinker	85 (40.1)	63 (29.7)	38 (17.9)	26 (12.3)	
Current/frequent drinker	33 (24.3)	30 (22.1)	22 (16.2)	51 (37.5)	
Comorbidities					
Zero	94 (41.6)	53 (23.4)	37 (16.4)	42 (18.6)	0.2175
One	66 (52.8)	26 (20.8)	17 (13.6)	16 (12.8)	
≥ Two	92 (42.0)	57 (26.0)	25 (11.4)	45 (20.6)	
Anatomic site					
Oropharynx	36 (38.7)	30 (32.3)	13 (14.0)	14 (15.0)	0.0215
Larynx	24 (38.1)	20 (31.8)	13 (20.6)	6 (9.5)	
Oral cavity	47 (52.8)	20 (22.5)	5 (5.6)	17 (19.1)	
Other/missing	145 (44.6)	66 (20.3)	48 (14.8)	66 (20.3)	

In the multivariable linear regression analyses, there were no statistically significant associations between current cannabis use and any of the FACT‐HN subscales (Table 3). Table 4 presents the findings from regression models examining associations between current illicit drug use and FACT‐HN subscales. Compared with patients denying current illicit drug use, those who were currently using illicit drugs had worse emotional well‐being (*β* = −1.32; 95% CI −2.45 to −0.20). However, there were no statistically significant associations between current illicit drug use and physical, social, and functional well‐being or head and neck cancer‐related symptom domains.

**TABLE 3:** Adjusted linear regression for cannabis use and FACT‐HN subscales.

	Adjusted *β* (95% CI)				
	Physical well‐being	Social well‐being	Emotional well‐being	Functional well‐being	Head and neck cancer
Cannabis use					
Never	Reference	Reference	Reference	Reference	Reference
Past	−0.06 (−1.15, 1.03)	−0.18 (−1.36, 1.00)	−0.67 (−1.65, 0.32)	0.27 (−1.23, 1.76)	0.82 (−0.97, 2.62)
Current	−0.42 (−1.75, 0.92)	−0.36 (−1.81, 1.09)	−1.13 (−2.34, 0.07)	−0.05 (−1.88, 1.79)	0.99 (−1.19, 3.19)
Missing	**1** **.36 (0.04, 2.67)**	0.55 (−0.88, 1.98)	−0.43 (−1.62, 0.76)	1.83 (0.02, 3.64)	1.73 (−0.43, 3.89)

*Note:* Model adjusted for age, gender, race, marital status, education level, insurance type, smoking status, alcohol use, comorbidities, and anatomic site. Bold values represents statistical significance at *p* < 0.05.

**TABLE 4:** Adjusted linear regression for ilicit drug use and FACT‐HN subscales.

	Adjusted *β* (95% CI)				
	Physical well‐being	Social well‐being	Emotional well‐being	Functional well‐being	Head and neck cancer
Current illicit drug use					
No	Reference	Reference	Reference	Reference	Reference
Yes	−1.04 (−2.29, 0.21)	−0.63 (−1.99, 0.72)	**−1.32 (−2.45, −0.20)**	−1.04 (−2.75, 0.68)	−0.07 (−2.13, 1.99)
Missing	1.15 (−0.08, 2.37)	0.54 (−0.79, 1.87)	−0.24 (−1.35, 0.87)	1.55 (−0.13, 3.24)	1.26 (−0.76, 3.28)

*Note:* Model adjusted for age, gender, race, marital status, education level, insurance type, smoking status, alcohol use, comorbidities, and anatomic site. Bold values represents statistical significance at *p* < 0.05.

## 4 Discussion

In this cross‐sectional study, we investigated the associations between cannabis use, illicit drug use, and patient‐reported QoL prior to starting cancer treatment in patients diagnosed with HNC. Current cannabis use was not independently associated with any of the four QoL domains (functional, physical, emotional, and social/familial well‐being). Current illicit drug use (i.e., cocaine, heroin, misuse of prescription medications) was independently and negatively associated with QoL in the emotional well‐being subscale.

The focus of previous research and intervention for substance use in patients with HNC has overwhelmingly focused on alcohol and tobacco use [1, 3, 7, 8, 9, 10, 12]. However, over the past several decades, changes in state legislation across the United States leading to decriminalizing recreational and medicinal cannabis use have mirrored changes in public perceptions of cannabis and its consumption [13]. With increased social acceptance and accessibility, understanding cannabis use in HNC care is of increased relevance. In a study assessing attitudes of patients with HNC actively undergoing treatment, 70% reported believing medical cannabis would help with symptoms during treatment [14]. Accordingly, clinicians may expect to be asked by patients with HNC about the potential role of cannabis in symptom management more frequently. Thus, the need for expanding the types of substances and methods of use screened for in the pretreatment period beyond tobacco and alcohol may be warranted for appropriate intervention aimed at improving patient well‐being, QoL, and, possibly, cancer treatment outcomes.

In the present study, we found that cannabis use was significantly associated with tobacco and alcohol consumption in bivariate analyses. While literature on the relationship between cannabis, alcohol, and tobacco use among patients with HNC is sparse, a retrospective study conducted by Xie et al. [15] found that among patients with HNC reporting marijuana use, a significantly smaller proportion of patients reported tobacco use and a greater‐than‐21 pack‐year smoking history. The same study found no significant association between marijuana and alcohol consumption among the same cohort [15]. Among the general cancer patient population, cannabis users were significantly more likely to smoke cigarettes at various points in the diagnosis and treatment courses and report concurrent substance use [16, 17]. Given the relationship between alcohol, tobacco, and potentially, cannabis, and the pathogenesis of HNC, there is a need to further explore how the use of one of these substances by patients with HNC can affect the likelihood to use another substance at various points in the diagnosis, treatment, and surveillance phases. This can inform the prognostication of the likelihood of recurrence and inform management and treatment plans. Furthermore, studies exploring the likelihood of the concurrent use of these substances can inform risk stratification and screening methods for patients reporting substance use. Lastly, several variables were found to affect the likelihood of concurrent medical cannabis and alcohol use, which are important to keep in mind when considering the potential role of medical cannabis in the management and treatment of patients with HNC [18].

Despite the growing interest in understanding cannabis use in patients with HNC, the current literature also remains limited regarding its association with QoL. In our study, we assessed association between cannabis use and QoL and found no relationship between the two after controlling for other factors in the multivariate analysis. Thus, the significant association with emotional well‐being on univariate analysis was likely due to other mediating factors, such as patients reporting cannabis use being younger and having a higher level of education. Prior studies investigating the effects of cannabis on QoL in patients with HNC demonstrated varying results. Zhang et al. [19] reported that in a case–control study of newly diagnosed patients with HNC, self‐reporters of cannabis use endorsed significantly better QoL compared to patients not using cannabis. Specifically, these patients reported decreased rates of anxiety, pain, nausea, and feelings of depression, and increased appetite and feelings of well‐being [19]. Our study probed all patients for cannabis use rather than relying on self‐report, which may introduce sampling bias. In other studies, cannabis use was associated with improved self‐reported symptoms of nausea, headache, and radiation therapy adverse effects, including weight loss, dysphagia, muscles spasms, and xerostomia, but no comparisons were made using validated QoL instruments [14, 20]. Conversely, some studies have found increased difficulty in pain management and increased weight loss during radiotherapy in patients with HNC who endorsed cannabis use [21, 22]. Further, there are some data indicating carcinogenesis concerns for cannabis use, particularly in HPV‐related HNC [23]. Finally, despite possible improvements in patient‐reported outcomes, disease‐free and overall survival were previously found to be negatively associated with cannabis use, further adding to the variability in findings on the role of cannabis [22]. Moving forward, research efforts should continue to focus on the impact of cannabis, in its various consumable forms (i.e., combustible and edible), on the well‐being of patients with HNC and overall outcomes pre‐, peri‐, and post‐treatment.

Understanding the effect of illicit drug use on patients with HNC is essential, especially considering the rising rates of prescription drug misuse in the United States [24]. We found that patients with HNC who used illicit drugs, including misused prescription medications, at the time of diagnosis had worse emotional well‐being than those who did not use illicit drugs. These findings are in accordance with the previous literature on relationships between illicit drug use and psychological well‐being in the general population, yet research on this subject remains limited when considering patients with HNC. In terms of the general population, one study found that a majority of patients entering treatment for opiate addiction exhibited depressive symptoms [25]. Another study found a strong association between illicit drug use and comorbid major depression [26]. When considering patients with HNC, previous research has demonstrated that current benzodiazepine misuse is associated with lower patient‐reported well‐being [27]. Whether poor emotional well‐being predisposes patients to an increased risk for illicit drug use in the form of a coping mechanism or if illicit drug use contributes to poor emotional well‐being as a sequalae of long term substance use in patients with HNC is yet to be determined.

### 4.1 Limitations

There are some limitations in the present study that are important to consider. First, given the stigma around substance use, patients may choose not to disclose their current or historical substance use to their healthcare teams. However, in the current study, the psych‐oncologist asked about all substance use with specific questions about illicit drug use using non‐judgmental language and clinical approach, thus improving likelihood of reporting use. Second, as a cross‐sectional study, no causative conclusions can be drawn. Third, there were variables that could have affected patients' quality of life such as stage and HPV status that were not included in the models due to their unavailability. Fourth, our study also only examined the period prior to treatment and did not examine continued substance use during active treatment or changes in QoL. Further study investigating the impact of continued substance use during treatment and into survivorship is required to understand its impact on longer term QoL among patients with HNC. Finally, the relationship between route of cannabis consumption (e.g., combustible vs. non‐combustible cannabis use) and patient QoL was not evaluated in this study, and it is possible that the route of consumption may have an impact on QoL and cancer treatment outcomes. Future research should examine route of consumption of cannabis, as there is concern for use of combustibles given the worse outcomes seen in continued combusted tobacco use throughout HNC treatment. Additionally, data on the exact duration, frequency, and dosage of certain illicit substances consumed was found to be unreliably reported by patients. Thus, broader categories relating to these variables, as outlined in the methods section, were used instead. However, these variables can introduce variance in the QoL effects of substance use and should be further explored with greater precision around “dose/unit” in future investigations. This area of research will further inform clinicians and patients about the risks and benefits of substance use, particularly cannabis use, during HNC treatment, allowing for improved shared decision‐making conversations and interventions, as is currently standard of care for alcohol and tobacco use.

## 5 Conclusion

Our findings suggest that illicit drug use, including prescription drug misuse, is independently and negatively associated with the emotional well‐being component of QoL in patients diagnosed with HNC prior to beginning cancer treatment. However, current cannabis use was not independently associated with patient QoL, despite being positively associated with emotional well‐being on univariate analysis. Given pretreatment QoL's role as both a predictive and prognostic factor for overall and disease‐free survival in patients with HNC, the QoL implications of all forms of substance use carry potential value in informing clinical decision making and appropriate treatment and management. Future research should longitudinally follow substance use across the treatment and survivorship spectrum to examine QoL and cancer outcomes.

## Author Contributions

**Eric Adjei Boakye:** conceptualization (equal), data curation (equal), formal analysis (equal), investigation (equal), methodology (equal), supervision (equal), validation (equal), writing – original draft (equal), writing – review and editing (equal). **Sami I. Nassar:** data curation (equal), investigation (equal), methodology (equal), validation (equal), writing – original draft (equal). **Suma J. Alzouhayli:** data curation (equal), investigation (equal), methodology (equal), validation (equal), writing – original draft (equal). **Amy M. Williams:** conceptualization (equal), data curation (equal), investigation (equal), methodology (equal), supervision (equal), validation (equal), writing – review and editing (equal). **Steven S. Chang:** investigation (equal), methodology (equal), validation (equal), writing – review and editing (equal). **Tamer A. Ghanem:** data curation (equal), investigation (equal), validation (equal), writing – review and editing (equal). **Marissa Gilbert:** data curation (equal), methodology (equal), validation (equal), writing – review and editing (equal). **Farzan Siddiqui:** data curation (equal), methodology (equal), validation (equal), writing – review and editing (equal). **Suhael Momin:** investigation (equal), methodology (equal), validation (equal), writing – review and editing (equal). **Vivian F. Wu:** investigation (equal), methodology (equal), validation (equal), writing – review and editing (equal). **Samantha H. Tam:** conceptualization (equal), data curation (equal), formal analysis (equal), investigation (equal), methodology (equal), validation (equal), writing – review and editing (equal).

## Ethics Statement

This study was approved by the Henry Ford Health Institutional Review Board and conducted in accordance with the Declaration of Helsinki.

## Consent

All respondents provided informed consent before filling out the survey.

## Conflicts of Interest

Dr. Samantha H. Tam received research funding from Genentech outside of the submitted work. Dr. Farzan Siddiqui received honorarium, travel reimbursement from Varian Medical System Inc. American College of Radiology, Castle Biosciences, and is a member on Varian Noona Medical Advisory Board. All other coauthors had no conflict to declare.

## Prior Meeting Presentation

The abstract of the manuscript was presented as a podium/oral presentation at the AHNS 11^th^ International Conference on Head & Neck Cancer, Jul 8–12, 2023; Montreal, QC, Canada.

## Data Availability

Analytic data will be provided by the corresponding author upon reasonable request.
